# Coffee and tea consumption in relation to inflammation and basal glucose metabolism in a multi-ethnic Asian population: a cross-sectional study

**DOI:** 10.1186/1475-2891-10-61

**Published:** 2011-06-02

**Authors:** Salome A Rebello, Cynthia H Chen, Nasheen Naidoo, Wang Xu, Jeannette Lee, Kee Seng Chia, E Shyong Tai, Rob M van Dam

**Affiliations:** 1 Life Sciences Institute, Centre for Life Sciences, National University of Singapore, 05-02, 28 Medical Drive, Singapore 117456; 2 Center for Molecular Epidemiology, National University of Singapore, #05-02, 28 Medical Drive, Singapore 117456; 3 Department of Epidemiology and Public Health, National University of Singapore, Yong Loo Lin School of Medicine, 16 Medical Drive, Singapore 117597; 4 Department of Medicine, National University of Singapore, 1E, Kent Ridge Road, NUHS Tower Block Level 10, Singapore 119228; 5 Department of Epidemiology and Public Health and Medicine, Yong Loo Lin School of Medicine, National University of Singapore Block MD3 #03-17, 16, Medical Drive, Singapore. 117597 and Department of Nutrition, Harvard School of Public Health, Boston, Massachusetts, 02115, USA

## Abstract

**Background:**

Higher coffee consumption has been associated with a lower risk of type 2 diabetes in cohort studies, but the physiological pathways through which coffee affects glucose metabolism are not fully understood. The aim of this study was to evaluate the associations between habitual coffee and tea consumption and glucose metabolism in a multi-ethnic Asian population and possible mediation by inflammation.

**Methods:**

We cross-sectionally examined the association between coffee, green tea, black tea and Oolong tea consumption and glycemic (fasting plasma glucose, HOMA-IR, HOMA-beta, plasma HbA1c) and inflammatory (plasma adiponectin and C-reactive protein) markers in a multi-ethnic Asian population (N = 4139).

**Results:**

After adjusting for multiple confounders, we observed inverse associations between coffee and HOMA-IR (percent difference: - 8.8% for ≥ 3 cups/day versus rarely or never; *P_trend _*= 0.007), but no significant associations between coffee and inflammatory markers. Tea consumption was not associated with glycemic markers, but green tea was inversely associated with plasma C-reactive protein concentrations (percent difference: - 12.2% for ≥ 1 cup/day versus < 1 cup/week; *P_trend _*= 0.042).

**Conclusions:**

These data provide additional evidence for a beneficial effect of habitual caffeinated coffee consumption on insulin sensitivity, and suggest that this effect is unlikely to be mediated by anti-inflammatory mechanisms.

## Background

The prevalence of Type-2 diabetes mellitus (Type-2 DM) is rising rapidly in Asian countries [1,2], and identifying lifestyle measures to improve insulin sensitivity and prevent Type-2 DM in these populations is particularly important. Data from multiple prospective cohort studies [3], including results from Asian populations [4,5], indicate that coffee consumption is associated with a lower risk of Type-2 DM. Results from several cross-sectional studies suggest that an association between coffee consumption and insulin sensitivity may contribute to this association [6,7]. Because coffee is a rich source of anti-oxidants [8], and sub-clinical inflammation has been implicated in the development of insulin resistance (IR) [9] coffee may improve insulin sensitivity by decreasing inflammation. Inflammatory markers including higher C-reactive protein (CRP) and lower adiponectin concentrations have been associated with insulin resistance [10,11].

However, studies on coffee consumption and inflammatory outcomes have yielded inconsistent results. Coffee consumption was associated with lower plasma CRP concentrations in four cross-sectional studies [12-15], with higher CRP concentrations in a Greek population [16], and with no change in CRP concentrations in a small clinical study [17]. Fewer studies have examined coffee consumption in relation to adiponectin concentrations. In cross-sectional studies in the U.S. [18] and Japan [19] coffee consumption was associated with higher adiponectin concentrations, whereas another study in Japan [20] observed no such association. In an intervention study, serum adiponectin concentration increased when participants consumed eight cups of coffee per day as compared to when they did not consume coffee [17]. These studies did not distinguish between high-molecular weight and lower molecular weight adiponectin that may have different metabolic effects [21].

There is less consistent evidence on the association between tea consumption and risk of Type-2 DM [3]. Data on the effects of tea in relation to glycemic parameters and inflammatory markers are inconclusive [22] and are mostly limited to green tea [23-26]. Since the putative bioactive compounds in tea vary by type, some of the discrepancies may be related to the type of tea consumed.

The main aim of this study was to evaluate consumption of coffee and three type of tea (black, green and Oolong) in relation to markers of inflammation, insulin sensitivity, and glycemia in a multi-ethnic population consisting of Chinese, Malays and Asian-Indians. We hypothesized that coffee and tea consumption are associated with lower IR and that this association can be partly explained by plasma adiponectin and CRP concentrations.

## Methods

### Study Population

In this study we used cross-sectional data from the Singapore Prospective study-2 (SP2) cohort. This study conducted between 2003-2007, was a follow-up of persons participating in four previous Singaporean population-based studies: the Thyroid and Heart Study [27], the National Health Survey-1992 [28], National University of Singapore Heart Study [29] and the National Health Survey-1998 [30] which were conducted between 1982 and 1998. These studies used a stratified random sampling method, disproportionate for ethnicity to increase representation of the minority Malay and Asian-Indian ethnic groups. The total participant pool derived from these studies consisted of 10,747 persons, of which 667 were not available (deceased, emigrated, errors in identity number recording) [31]. For the SP2 study, an interviewer-administered questionnaire was completed during a home visit by 7744 participants, and 5163 of these participants attended a clinic visit. Data on demographic and lifestyle characteristics and medical history were collected during the interview and fasting blood samples were obtained during the clinic visit for measuring biochemical markers [32]. For the present analyses, we only included people who attended the clinic visit (n = 5163). We excluded data from participants who reported pre-existing diabetes (n = 494), cardiovascular disease (n = 213) and current cancer (n = 56) or those for whom these data were missing (n = 76). We also excluded people who reported their race as 'other' (n = 3) and those who were pregnant (n = 2). Of the remaining 4417 people, 220 had an implausible energy intake (> 7000 or < 400 kcal/day, or who had an energy intake to energy expenditure ratio that fell in extreme 2.5 percentile range) and 35 people had reported changing their beverage intake in the month preceding the questionnaire. After excluding these people, 22 had missing covariate data. After these exclusions our final dataset comprised of 4139 participants. The study was approved by the Singapore General Hospital and the National University Hospital Institutional Review Boards.

### Dietary data

Dietary intake data during the month preceding the interview was assessed using a semi-quantitative 169-item validated food frequency questionnaire that is used in the National Nutrition Surveys [33]. Participants were asked to estimate their frequency of consuming each food group, based on a standard portion size specific for that food group. Participants could report consumption per day, per week or per month, or could report to never or rarely consume a food. Nutrient intakes were computed by the Health Promotion Board of Singapore, using their in-house database.

Data on coffee intake were obtained by asking participants about the habitual amount of coffee consumed. Participants could select one of seven responses ranging from 'never/rarely' to '10 or more cups per day', with 1 cup being defined as a "standard coffee-shop cup" of 215 ml. Similar questions were asked about green, Oolong and black tea. Coffee intake was grouped into four categories (never/rarely, < 1 cup/day, 1-2 cups/day and ≥ 3 cups per day) and tea intake into 3 categories (< 1 cup/week, 2-6 cups/wk, ≥ 1 cup per day) for data analyses to avoid categories with small numbers. The questionnaire also elicited information on the amount of sugar and the type and amount of milk/cream added to coffee and tea. Although data on type of coffee were not collected, decaffeinated coffee intake is very low in the Singapore population [4].

### Assessment of covariates

Height was measured using a wall mounted measuring tape and weight was measured using a digital scale. BMI was computed as weight (kg) divided by height (m^2^). Physical activity level and smoking status was assessed from the questionnaire and alcohol intake was obtained from the FFQ. Total physical activity was assessed using a validated questionnaire on household, occupational, leisure-time and transport activity [34]. Participants who used medications to lower cholesterol or triglycerides or increase HDL-C concentrations, or who reported having been diagnosed with hypercholesterolemia were considered to have a history of dyslipidemia. Participants who used anti-hypertensive medication or reported having been diagnosed by a physician with hypertension were considered to have a history of hypertension.

### Laboratory Analyses

Fasting plasma samples were analyzed for glucose using enzymatic methods (ADVIA 2400, Siemens, Germany). Fasting blood samples were used to measure HbA1c using high pressure liquid chromatography on a Biorad Variant II analyzer (Bio-rad laboratories, Hercules, CA, USA). Fasting serum samples were analyzed for insulin, using micro-particle enzyme immunoassay (Abbott AXSYM, Abbott Laboratories, Chicago, IL), for high-sensitivity C-reactive protein, using an immunoturbidimetric assay (Roche Diagnostics, Rotkreuz, Switzerland), and for total and high molecular weight (HMW) adiponectin using an enzyme linked immunosorbent assay (Sekisui Medical Co Ltd, Japan). The intra and inter batch coefficent of variations percent were as follows; glucose (2.5, 6.6), insulin (4.0, 4.5), HbA1c (0.0-2.0, 0.85-1.54), total adiponectin (18.1, 15.9) HMW adiponectin (6.8, 18.3) and CRP (intraassay precision, 0.6%-1.3%; interassay precision, 2.3%-3.1%).

### Statistical Analyses

All response variables were transformed using natural logarithms to approximate normality. The means and 95% confidence intervals (CIs) were then exponentiated to obtain geometric means and 95% CIs. Values for response variables that were more than four standard deviations from the mean were considered outliers and were excluded from the analyses. The HOMA-IR index was computed as fasting plasma glucose mmol/L × fasting serum insulin (mU/L)/22.5 and the HOMA-beta index was computed as 20 × fasting serum insulin (mU/L)/fasting plasma glucose (mmol/L -3.5) [35]. Participant characteristics were compared across categories of coffee and tea using Kruskal-Wallis (for non-normally distributed continuous variables), analysis of variance (for normally distributed continuous variables) or the chi-squared test (for categorical variables). Multiple linear regression analyses were performed using HOMA-IR, HOMA-beta, plasma glucose, HbA1c, plasma insulin, CRP, total adiponectin and HMW adiponectin as dependant variables and coffee or tea as independent variables. Linear estimators were fitted using two multivariate models: Model 1:adjusted for age, sex and ethnicity Model 2: further adjusted for BMI (kg/m^2^), physical activity (kcal/week), education level, alcohol consumption, cigarette smoking, history of dyslipidemia, history of hypertension and dietary factors i.e. energy intake (kcal), fiber (per 1000 kcal), cholesterol (per 1000 kcal), PUFA (% energy), MUFA (% energy), SFA (% energy), coffee (for tea analyses) and tea (for coffee analyses). HOMA-beta, as a marker for beta cell function must be considered within the context of IR, because insulin sensitivity regulates insulin secretion [36]. Models for HOMA-beta were therefore further adjusted for HOMA-IR. Residual plots and Cooks distance test (Cooks D < 1.0) was used to assess if there were influential points in the multivariate models [37]. Influential points were not observed with a maximum Cooks Di of 0.03. For trend tests, the median number of cups of coffee or tea consumed in each coffee category or each tea category was treated as a continuous variable. We tested for interactions with overweight status by including multiplicative interaction terms in the model after adjusting for all potential confounders. To evaluate whether additions to coffee modified observed associations, stratified analyses were performed for groups of participants that reportedly added or did not add milk to coffee, and for those that reportedly added or did not add sugar to beverages excluding individuals who used the other type of coffee. Because strong correlations were noted between HOMA-IR and fasting insulin (r_spearman _= 0.98) and between total and HMW adiponectin (r_spearman _= 0.94), results are not presented for fasting insulin and total adiponectin. All data were analyzed using SPSS v11 (SPSS Inc. Chicago, IL). P-values < 0.05 were considered statistically significant

## Results

### Participant Characteristics

Coffee drinkers were more likely to be older, male, non-tea drinkers, alcohol consumers, cigarette smokers with higher physical activity, a lower education level, and less healthy dietary choices **(**Table 1**)**. Most coffee drinkers consumed their coffee with milk/cream (71.4%) and sugar (63.0%) Characteristics of tea drinkers varied by type of tea consumed (Additional file 1**Table S1)**. Black tea and Oolong tea drinkers were more likely to be older, male and have higher BMI. Green tea consumers were more likely to be younger. Chinese were more likely to consume Oolong or green tea, and less likely to consume black tea than Indians or Malay. Black tea consumption was associated with less health conscious dietary intakes, and more physical activity. Oolong and green tea drinkers were more likely to consume alcohol. Regardless of tea type, tea drinkers were more likely to be highly educated and to have known hypertension.

**Table 1:** Characteristics of study participants across categories of coffee consumed^a^

	Total	Never or rarely *(n = 1202)*		< 1 cup per day *(n = 475)*	1-2 cups per day *(n = 2118)*	≥ 3 cups per day *(n = 344)*		*P*-value
Age (y)	48.8 ± 11.3	46.9 ± 11.7		47.1 ± 11.7	50.2 ± 11.0	49.2 ± 9.9		<0.001
BMI (kg/m^2^)	23.2 (20.8 - 26.0)	23 (20.4 - 26.09)		23.2 (20.5 - 25.9)	23.3 (20.9 - 26.1)	23.4(21.5 - 26.0)		0.0317
Females, n (%)	2218 (53.6)	739 (61.5)		265 (55.8)	1112 (52.5)	102 (29.7)		<0.001
Ethnicity, n (%)								0.117
Chinese	2868 (69.3)	793 (66.0)	337 (70.9)		1503 (71.0)	235 (68.3)		
Malay	698 (16.9)	228 (19.0)	77 (16.2)		332 (15.7)	61 (17.7)		
Indian	573 (13.8)	181 (15.1)	61 (12.8)		283 (13.4)	48 (14.0)		
Primary education and below, n (%)	998 (24.1)	236 (19.6)	99 (20.8)		569 (26.9)	94 (27.3)		< 0.001
Alcohol consumption, n (%)								0.003
Non-drinker	3371 (81.4)	1016 (84.5)	379 (79.8)		1711 (80.8)	265 (77.0)		
< 1 serving per day	690 (16.7)	175 (14.6)	88 (18.5)		356 (16.8)	71 (20.6)		
≥ 1 serving per day	78 (1.9)	11 (0.9)	8 (1.7)		51 (2.4)	8 (2.3)		
Current cigarette smoking, n (%)	485 (11.7)	76 (6.3)		41 (8.6)	262 (12.4)		106 (30.8)	< 0.001
Black tea (cups/week)^b^	4.4 ± 6.0	5.5 ± 7.0		4.6 ± 5.5	3.7 ± 5.2		4.0 ± 6.9	< 0.001
Chinese tea (cups/week)^b^	2.1 ± 5.2	2.1 ± 5.5		2.8 ± 6.1	2.0 ± 4.7		1.8 ± 5.6	< 0.001
Green tea (cups/week)^b^	1.6 ± 3.9	1.7 ± 4.0		2.0 ± 4.2	1.4 ± 3.8		1.4 ± 3.9	< 0.001
Physical activity (kcal/week)	3694 (1922 - 6728)	3736 (1810 - 7086)		3353 (1885 - 5999)	3725 (1952 - 6605)		4160(2075 - 8055)	0.031
% energy from SFA	11.1 ± 2.8	11 ± 2.9		10.9 ± 2.7	11 ± 2.8		11.8 ± 2.8	< 0.001
% energy from MUFA	9.7 ± 2.6	9.8 ± 2.7		9.8 ± 2.6	9.6 ± 2.6		9.5 ± 2.6	0.115
% energy from PUFA	5.3 (4.2 - 7.3)	5.6 (4.2 - 7.6)		5.4 (4.2 - 7.2)	5.3 (4.2 - 7.2)		4.7 (3.8 - 6.4)	< 0.001
Fiber (g per 1000 kcal)	10.5 (9.1 - 12.3)	11.0 (9.3 - 12.8)		10.6 (9.3 - 12.4)	10.5 (9.1 - 12.2)		9.2 (8.0 - 10.8)	< 0.001
Cholesterol (mg per 1000 kcal)	113.6 (88.0 -140.5)	112.2 (86.4 - 141.9)		110.5 (86.3 - 141.3)	114.3 (88.7 - 138.2)		116.9 (93.0 - 146.6)	0.016
History of hypertension, n (%)	710 (17.2)	214 (17.8)		80 (16.8)	373 (17.6)		43 (12.5)	0.114
History of dyslipidemia, n (%)	1139 (27.5)	311 (25.9)		117 (24.6)	609 (28.8)		102 (29.7)	0.111

^a ^Abbreviations: SFA; saturated fatty acid, MUFA; monounsaturated fatty acids, PUFA; polyunsaturated fatty acids. Data are presented as mean ± SD, median (IQR) or n (%) unless otherwise specified.

^b ^Median values were assigned to each category of coffee/tea as follows: never/rarely; 0, < 1 cup/week; 0.4, > than 1 cup/wk but < 1cup/d; 4;1-2 cups/d; 10.5; 3-5 cups/d; 28; 6-9 cups/day; 52.5 and 10 or more cups/day; 70. Means and SD are presented in the tables; due to skewed data the Kruskal-Wallis test was used to test for differences between categories.

### Associations between coffee and glycemic and inflammatory markers

No associations between coffee and glycemic markers were noted in age, sex and ethnicity adjusted models **(**Table 2**)**. However, after adjusting for BMI, coffee consumption was inversely associated with IR (*P_trend_= *0.021), an association that remained after further adjustment for dietary covariates and history of hypertension and dyslipidemia (percent difference: - 8.8% for ≥ 3 cups per day versus rarely or never; *P_trend _*= 0.007). This association did not differ significantly by ethnicity (*P_interaction _*= 0.769) or overweight status (BMI < or ≥ 25 kg/m^2^) (*P_interaction _*= 0.374). Coffee consumption was not associated with plasma CRP and adiponectin in any of the models (Table 2). Consistent with this observation, the association between coffee consumption and HOMA-IR was not substantially weaker after further adjustment for inflammatory markers (percent difference = -7.7% for ≥ 3 cups/day versus rarely or never; *P_trend _*= 0.021) (Table 2). The association between coffee and IR was similar for coffee with added milk (percent difference = - 8.76% for ≥ 3 cups/day versus rarely or never; *P_trend _*= 0.0078) and coffee without added milk (percent difference = - 8.56%; *P_trend _*= 0.1943; *P*_interaction _= 0.9165) and for added sugar (percent difference = - 7.23%; *P_trend _*= 0.0452) and no added sugar (percent difference = - 12.21%; *P_trend _*= 0.007; *P*_interaction _= 0.6041).

**Table 2:** Geometric means (95% CI) of glycemic and inflammatory parameters by categories of coffee consumption^a^

	Never or rarely	< 1 cup/day	1-2 cup(s)/day		≥ 3 cups/day	*P *trend^b ^
Fasting plasma glucose (mmol/L)						
Model-1^c^	4.83 (4.79 - 4.86)	4.81 (4.75 - 4.86)	4.86 (4.83 - 4.89)		4.82 (4.76 - 4.88)	0.554
Model-2	4.76 (4.69 - 4.82)	4.73 (4.66 - 4.81)	4.78 (4.72 - 4.84)		4.74 (4.66 - 4.82)	0.916
HOMA-IR						
Model-1	1.58 (1.52 - 1.65)	1.54 (1.44 - 1.64)	1.56 (1.51 - 1.62)		1.53 (1.42 - 1.64)	0.449
Model-2	1.47 (1.38 - 1.57)	1.41 (1.30 - 1.52)	1.41 (1.32 - 1.50)		1.34 (1.23 - 1.46)	0.007
Model-3^d^	1.43 (1.34 - 1.53)	1.36 (1.26 - 1.47)	1.36 (1.28 - 1.45)		1.32 (1.22 - 1.44)	0.021
HOMA-beta						
Model-1	104.68 (101.2 - 108.28)	105.57 (100.27 - 111.15)	100.54 (97.81 - 103.35)	106.42 (100.24 - 112.98)		0.715
Model-2	106.24 (99.34 - 113.63)	106.15 (98.43 - 114.47)	101.9 (95.64 - 108.57)	107.02 (98.29 - 116.51)		0.591
HbA1c %						
Model-1	5.79 (5.76 - 5.83)	5.72 (5.67 - 5.78)	5.78 (5.76 - 5.81)		5.8 (5.74 - 5.86)	0.567
Model-2	5.75 (5.69 - 5.82)	5.69 (5.62 - 5.76)	5.72 (5.66 - 5.78)		5.72 (5.64 - 5.8)	0.375
Adiponectin (high molecular weight) µg/ml						
Model-1	1.03 (0.98 - 1.08)	1.04 (0.97 - 1.12)	1.02 (0.98 - 1.06)		1.03 (0.95 - 1.12)	0.953
Model-2	0.99 (0.91 - 1.08)	1.02 (0.92 - 1.12)	1.00 (0.92 - 1.08)		1.04 (0.93 - 1.16)	0.347
CRP (mg/L)						
Model-1	1.42 (1.32 - 1.52)	1.59 (1.43 - 1.77)	1.49 (1.41 - 1.58)		1.59 (1.41 - 1.81)	0.139
Model-2	1.31 (1.16 - 1.49)	1.43 (1.25 - 1.65)	1.28 (1.14 - 1.44)		1.23 (1.05 - 1.44)	0.185

^a ^HOMA-IR, homeostatic model assessment-insulin resistance; HOMA-beta, homeostatic model assessment-beta cell function; CRP, C-reactive protein. Numbers of participants differ across outcomes because participants with outlier values (response values > 4 SD from mean) were excluded. These numbers across the 4 categories of coffee consumption are fasting plasma glucose: 1187,461,2084,342; HOMA-IR: 1195,468,2102,343; HOMA-beta: 1187, 468, 2097, 343; HbA1c: 967, 388, 1717, 281; HMW adiponectin: 1176, 452, 2056, 333 and CRP: 1168, 450, 2046, 331.

^b ^*P*-values were obtained from multiple linear regression models with median cups of coffee per week (0, 4,10.5 and 28) as predictors and log transformed parameters as dependant variables.

^c ^Model-1: adjusted for age (years), sex and ethnicity (Chinese, Malay, Indian).

Model-2: adjusted for Model-1 covariates (above) and BMI (kg/m^2^), physical activity level (kcal/week), education level (primary, secondary, polytechnic/diploma and university), alcohol level (non-drinkers, < 1 serving/day, and ≥ 1 serving/day), cigarette smoking (never-smokers, ex-smokers, current smokers < 10 cigarettes/d, and current smokers ≥ 10 cigarettes/d), history of dyslipidemia (yes/no), history of hypertension (yes/no), and dietary confounders i.e. energy intake (kcal), fiber (per 1000 kcal), cholesterol (per 1000 kcal), PUFA (% energy), MUFA (% energy), SFA (% energy), green, black, Oolong tea (never/< 1cup per week, 2-6 cups per week, ≥ 1 cup(s) per day). HOMA-beta models were further adjusted for HOMA-IR.

^d ^Model-3 was further adjusted for CRP and adiponectin

### Associations between tea and glycemic and inflammatory markers

Black, Oolong, and green tea consumption were not significantly associated with glycemic markers in fully adjusted models (Tables 3, 4 and 5**)**. Tea consumption was not significantly associated with inflammatory markers in age, sex and ethnicity adjusted models. However after adjustment for other potential confounders, green tea consumption was significantly associated with lower CRP concentrations (percent difference: - 12.2% for ≥ 1 cup/day versus < 1 cup/week; *P_trend _*= 0.042) (Table 5). This association was not modified by BMI status (*P*_interaction _= 0.1032) or ethnicity (*P*_interaction _= 0.939). Few people consumed three or more cups of a given tea-type (N = 32, 71 and 98 for green, Oolong and black tea respectively), making it less meaningful to look at higher categories of tea intake. When we summed tea intake across the different types of tea, we found no significant associations between tea consumption and metabolic markers **(**Additional file 1**Table S2)**.

**Table 3:** Geometric means (95% CI) of glycemic and inflammatory parameters by categories of black tea consumption^a^

	never or < 1 cup per week	2-6 cups per week	≥ 1 cup(s) per day	*P *trend^b^
Fasting plasma glucose (mmol/L)				
Model-1^c^	4.86 (4.83 - 4.89)	4.81 (4.77 - 4.86)	4.83 (4.80 - 4.86)	0.144
Model-2	4.78 (4.72 - 4.84)	4.73 (4.66 - 4.80)	4.75 (4.68 - 4.81)	0.115
HOMA-IR				
Model-1	1.55 (1.50 - 1.60)	1.49 (1.42 - 1.57)	1.61 (1.55 - 1.67)	0.141
Model-2	1.43 (1.34 - 1.52)	1.35 (1.25 - 1.45)	1.44 (1.35 - 1.54)	0.579
HOMA-beta				
Model-1	101.07 (98.18 - 104.04)	105.48 (101.04 - 110.13)	103.83 (100.59 - 107.17)	0.169
Model-2	103.34 (96.79 - 110.34)	107.12 (99.69 - 115.10)	105.49 (98.70 - 112.76)	0.320
HbA1c %				
Model-1	5.79 (5.76 - 5.82)	5.75 (5.71 - 5.80)	5.79 (5.76 - 5.82)	0.987
Model-2	5.73 (5.67 - 5.80)	5.71 (5.64 - 5.78)	5.72 (5.66 - 5.79)	0.698
Adiponectin: high-molecular weight (µg/ml)				
Model-1	1.05 (1.01 - 1.09)	1.02 (0.96 - 1.08)	1 (0.96 - 1.05)	0.110
Model-2	1.03 (0.94 - 1.12)	1.01 (0.92 - 1.11)	1 (0.92 - 1.09)	0.327
CRP (mg/L)				
Model-1	1.50 (1.41 - 1.59)	1.46 (1.33 - 1.60)	1.49 (1.40 - 1.59)	0.929
Model-2	1.34 (1.19 - 1.52)	1.28 (1.12 - 1.46)	1.32 (1.17 - 1.5)	0.730

^a ^HOMA-IR, homeostatic model assessment-insulin resistance; HOMA-beta, homeostatic model assessment-beta cell function; CRP, C-reactive protein. Numbers of participants differ across outcomes because participants with outlier values (response values > 4 SD from mean) were excluded. Numbers were as follows: fasting plasma glucose: 2130, 723, 1221 HOMA-IR: 2148, 727, 1233, HOMA-beta: 2143, 725, 1227, HbA1c: 1758, 587, 1008 HMW adiponectin: 2097, 705, 1215; and CRP: 2082, 700, 1213.

^b ^*P*-values were obtained from multiple linear regression models with median cups of tea per week (0, 4,10.5) as predictors and log transformed parameters as dependant variables.

^c ^Model-1: adjusted for age (years), sex and ethnicity (Chinese, Malay, Indian).

Model-2: adjusted for Model 1 covariates (above) and BMI (kg/m^2^), physical activity level (kcal/week), education level (primary, secondary, polytechnic/diploma and university), alcohol level (non-drinkers, < 1 serving/day, and ≥ 1 serving/day), cigarette smoking (never-smokers, ex-smokers, current smokers < 10 cigarettes/d, and current smokers ≥ 10 cigarettes/d), history of dyslipidemia (yes/no), history of hypertension (yes/no), and dietary confounders i.e. energy intake (kcal), fiber (per 1000 kcal), cholesterol (per 1000 kcal), PUFA (% energy), MUFA (% energy), SFA (% energy), coffee (never/rarely, < 1 cup per day, 1-2 cup(s) per day, ≥ 3 cups/day) and alternate teas (never or < 1 cup per week, 2-6 cups per week, ≥ 1 cup(s) per day). HOMA-beta models were further adjusted for HOMA-IR.

**Table 4:** Geometric means (95% CI) of glycemic and inflammatory parameters by categories of Oolong tea consumption^a^

	never or < 1 cup per week	2-6 cups per week		≥ 1 cup(s) per day	*P *trend^b^
Fasting plasma glucose (mmol/L)					
Model-1^c^	4.84 (4.82 - 4.86)	4.82 (4.77 - 4.87)	4.86 (4.81 - 4.92)		0.591
Model-2	4.75 (4.69 - 4.82)	4.75 (4.68 - 4.82)	4.76 (4.68 - 4.83)		1.000
HOMA-IR					
Model-1	1.56 (1.51 - 1.60)	1.56 (1.47 - 1.65)	1.68 (1.57 - 1.79)		0.044
Model-2	1.41 (1.32 - 1.50)	1.40 (1.30 - 1.51)	1.41 (1.30 - 1.53)		0.940
HOMA-beta					
Model-1	102.50 (100.2 - 104.85)	105.31 (100.28 - 110.60)	103.70 (98.02 - 109.71)		0.511
Model-2	103.83 (97.48 - 110.60)	106.22 (98.69 - 114.32)	105.89 (97.91 - 114.52)		0.428
HbA1c %					
Model-1	5.78 (5.76 - 5.81)	5.76 (5.71 - 5.80)	5.81 (5.76 - 5.87)		0.461
Model-2	5.72 (5.66 - 5.78)	5.71 (5.64 - 5.78)	5.73 (5.66 - 5.81)		0.685
Adiponectin- high molecular weight (µg/ml)					
Model-1	1.03 (1.00 - 1.06)	1.04 (0.97 - 1.11)	0.96 (0.89 - 1.03)		0.105
Model-2	1.01 (0.93 - 1.09)	1.04 (0.94 - 1.14)	0.99 (0.89 - 1.09)		0.829
CRP (mg/L)					
Model-1	1.48 (1.41 - 1.55)	1.46 (1.32 - 1.62)	1.63 (1.45 - 1.83)		0.166
Model-2	1.29 (1.14 - 1.45)	1.30 (1.13 - 1.49)	1.35 (1.17 - 1.56)		0.397

^a ^HOMA-IR, homeostatic model assessment-insulin resistance; HOMA-beta, homeostatic model assessment-beta cell function; CRP, C-reactive protein. Numbers of participants differ across outcomes because participants with outlier values (response values > 4 SD from mean) were excluded. Numbers were as follows: fasting plasma glucose: 3058, 585, 431 HOMA-IR: 3087, 588, 433 HOMA-beta: 3080, 588, 427 HbA1c: 2493, 482, 378 HMW adiponectin: 3020, 572, 425 CRP: 3012, 564, 419.

^b, c ^For these footnotes please refer to Table 3

**Table 5:** Geometric means (95% CI) of glycemic and inflammatory parameters by categories of green tea consumption^a^

	never or < 1 cup per week	2-6 cups per week	≥ 1 cup(s) per day	*P *trend^b^
				
Fasting plasma glucose (mmol/L)				
Model-1^c^	4.84 (4.82 - 4.86)	4.83 (4.78 - 4.88)	4.87 (4.80 - 4.93)	0.575
Model-2	4.75 (4.69 - 4.81)	4.75 (4.68 - 4.82)	4.76 (4.68 - 4.85)	0.687
HOMA-IR				
Model-1	1.55 (1.51 - 1.60)	1.60 (1.51 - 1.69)	1.65 (1.53 - 1.78)	0.086
Model-2	1.40 (1.31 - 1.49)	1.43 (1.33 - 1.54)	1.39 (1.28 - 1.51)	0.920
HOMA-beta				
Model-1	103.02 (100.69 - 105.4)	103.13 (98.34 - 108.15)	100.17 (93.93 - 106.83)	0.465
Model-2	107.68 (101.1 - 114.69)	105.65 (98.22 - 113.64)	102.65 (94.44 - 111.58)	0.149
HbA1c %				
Model-1	5.78 (5.76 - 5.81)	5.75 (5.7 - 5.79)	5.81 (5.74 - 5.87)	0.943
Model-2	5.73 (5.67 -5.79)	5.7 (5.63 - 5.77)	5.74 (5.66 - 5.82)	0.883
Adiponectin High molecular weight (μg/ml)				
Model-1	1.03 (1.00 - 1.06)	1.02 (0.96 - 1.09)	0.97 (0.89 - 1.05)	0.162
Model-2	1.01 (0.93 - 1.09)	1.02 (0.92 - 1.11)	1.01 (0.91 - 1.12)	0.920
CRP (mg/L)				
Model-1	1.50 (1.43 - 1.57)	1.45 (1.31 - 1.60)	1.42 (1.24 - 1.62)	0.318
Model-2	1.39 (1.23 - 1.56)	1.34 (1.17 - 1.54)	1.22 (1.04 - 1.42)	0.042

^a ^HOMA-IR, homeostatic model assessment-insulin resistance; HOMA-beta, homeostatic model assessment-beta cell function; CRP, C-reactive protein. Numbers of participants differ across outcomes because participants with outlier values (response values > 4 SD from mean) were excluded. Numbers were as follows: fasting plasma glucose: 3169, 605, 300 HOMA-IR: 3193, 612, 303, HOMA-beta: 3188, 608, 299 HbA1c: 2586, 504, 263 HMW adiponectin: 3112, 602, 303 and CRP: 3091, 603, 301.

^b, c ^For these footnotes please refer to Table 3

## Discussion

In this study, in a multi-ethnic Singaporean population, coffee consumption was inversely associated with IR independent of plasma CRP or adiponectin concentrations. This association appeared to be consistent for overweight and non-overweight participants and for Chinese, Malay, and Asian Indian participants. We also noted an inverse relationship between green tea and plasma CRP concentrations, but found no evidence for an association between tea consumption and basal glucose metabolism.

To the best of our knowledge this is the first study to examine the extent to which inflammatory markers such as CRP and adiponectin explain the association between coffee consumption and IR. Data from other clinical and cross-sectional studies suggest a beneficial effect of coffee consumption on insulin sensitivity. However few studies have examined coffee in relation to insulin sensitivity [6] or inflammation [13,15,19,20] in Asian populations and these studies were all conducted in Japan. These cross-sectional studies found inverse associations between coffee and serum CRP concentrations [13,15], and either no association [20] or a direct association [19] with serum adiponectin concentrations. Both adiponectin and CRP have been associated with Type-2 DM risk [38,39]. Adiponectin can also decrease IR, independent of its anti-inflammatory properties, via the AMP-activated protein kinase pathway [40].

Although we observed an inverse association between coffee consumption and IR, we found no significant associations between coffee intake and inflammatory markers and these markers did not explain the association between coffee and IR. These results suggest that the putative protective effect of coffee consumption against the development of Type-2 DM, which has been previously reported in Singapore Chinese [4], is at least partly mediated by its effects on IR. However, this inverse association between coffee consumption and IR is probably not mediated by anti-inflammatory effects. Nevertheless, there are some caveats that are worth considering. First, although CRP is a well-established marker of systemic inflammation [41], it is possible that coffee alters other measures of inflammation that were not examined in this study. Second, the relatively low amounts of coffee consumed in this population, may have been insufficient to exert a biological effect on CRP and adiponectin. In a cross-sectional study of U.S. women, only consuming four or more cups of coffee per day, but not lower consumption, was associated with higher adiponectin concentrations [18]. However, the literature on this topic is not consistent with a few other studies suggesting associations with concentrations of CRP and adiponectin at intakes as low as one cup per day [13,19].

Apart from its anti-inflammatory effects, phenolic compounds in coffee have been postulated to affect glucose metabolism through various mechanisms including intestinal glucose absorption and incretin secretion [8], and reduction of hepatic triglyceride accumulation [8]. Consistent with several intervention studies, no associations were noted between black or green tea, and fasting glycemic parameters in this investigation [23,24,26]. In contrast, a Japanese intervention study, noted reduced fasting plasma glucose and fructosamine concentrations in diabetic participants who consumed 1 L per day of Oolong tea for four weeks [42]. The difference in diabetes status or amount of tea consumed may have contributed to the difference in results as compared with our study.

Tea consumption was not associated with adiponectin concentrations in our study. This is consistent with studies that showed no associations between green tea and plasma adiponectin concentration even at relatively high intake levels of four cups/day [19,43], but contrasts with one randomized trial in which consumption of one L of Oolong tea per day increased adiponectin concentrations in 22 persons with coronary artery disease [44].

The inverse association between green tea and CRP is a finding that stands in contrast to several other studies which observed no such association [25,43,45,46]. However, these studies were all clinical trials that examined either the acute [46] or short-term (4-8 weeks) [25,43,45] effects of green tea intake. A longer duration may be needed for the beneficial effects of green tea on CRP to be manifested. Interestingly, we found no associations between black tea and glycemic parameters or inflammatory markers. These findings were inconsistent with those of a prospective cohort study in Singaporean Chinese that found an inverse association between black tea consumption and Type-2 DM risk [4]. It is possible that black tea alters post-prandial aspects of glucose metabolism, which were not examined in this study.

The large multi-ethnic study population and detailed assessments of potential confounders are strengths of this study. Because of the cross-sectional design, the sequence of events cannot be inferred from this study. However, we excluded people with known diseases who may have exhibited differential recall of lifestyle exposures or were on medications that may have obscured the effects of coffee or tea on the markers of interest. Also, measurement error in the assessment of lifestyle exposures is unavoidable and makes the possibility of residual confounding a concern. However, as coffee consumption was associated with less health-conscious lifestyle behaviors, it is less likely that un-measured confounders would weaken the inverse association between coffee and IR. Green tea intake was associated with some favorable lifestyle behaviors and higher education, and it is thus possible that green tea may have served as a proxy for an unmeasured or imperfectly measured beneficial exposure in this study.

## Conclusions

These data suggest a beneficial effect of coffee consumption on insulin sensitivity in Asians at modest levels of consumption. This association did not appear to be mediated by anti-inflammatory mechanisms, and other pathways should be considered for mechanistic studies.

## Abbreviations

CRP: C-reactive protein; FPG: Fasting plasma glucose; HMW: High molecular weight; HOMA-beta: Homeostatic model assessment of beta cell function; HOMA-IR: Homeostatic model assessment of insulin resistance; IR: Insulin resistance; SP-2: Singapore Prospective study-2 cohort Type-2 DM: Type-2 Diabetes Mellitus;

## Competing interests

Tai E. Shyong. has served on advisory boards for Merck Sharp and Dohme (I.A.) Corp, Novo Nordisk Pharma (Singapore) Pte Ltd, Astra Zeneca (Singapore) Pte Ltd and Bristol Myers Squibb (Singapore) Pte Ltd., and has received honoraria for speaking engagements from Merck Sharp and Dohme (I.A.) Corp, Abbott Manufacturing Singapore Private Limited and Unilever Colworth. The other authors declare no competing interests.

## Authors' contributions

EST and JL designed the cohort study and directed its implementation. CHC and WX conducted the data analysis. SAR conducted the literature review, helped in data analysis and drafted the manuscript. NN helped in data cleaning and conducted technical review. KSC conceived of the research question and guided data analyses. RMVD guided data analyses and interpretation and led manuscript writing. All authors read and approved the final manuscript.

## Supplementary Material

Additional file 1 **Additional Tables**. Table S1: This table contains information on participant characteristics across categories of tea intake. Table S2: This table contains information on the associations between total tea consumption and metabolic markers. Click here for file
