# Supplementary material for: Coffee and tea consumption in relation to inflammation and basal glucose metabolism in a multi-ethnic Asian population: a cross-sectional study
Source: Nutr J. 2011 Jun 2;10:61. doi: 10.1186/1475-2891-10-61 (PMC3130641; doi:10.1186/1475-2891-10-61)
Supplement: Additional file 1 — Additional Tables. Table S1: This table contains information on participant characteristics across categories of tea intake. Table S2: This table contains information on the associations between total tea consumption and metabolic markers. [file 1475-2891-10-61-S1.PDF]

Table S1: Characteristics of study participants across categories of tea consumed [a]

	Black Tea never / <1 cup per week n = 2612	Black Tea ≥ 1 cup(s) per day n =1246	P-value	Oolong Tea never / <1 cup per week n = 3113	Oolong Tea ≥1 cup(s) per day n = 436	P-value	Green Tea never / <1 cup per week n = 3217	Green Tea ≥1 cup(s) per day n =309	P-value
Age (y)	49.0 ±11.6	49.6 ±10.7	<0.001	48.6 ±11.5	51.1 ± 10.8	<0.001	49.5 ±11.3	47.9 ±10.2	<0.001
BMI (kg/m^2)	22.9 (20.5-25.6)	24.0 (21.5-26.8)	<0.001	23.3 (20.8-26.1)	23.4 (21.2-25.9)	0.006	23.3 (20.8-26.1)	23.5 (21.1-25.9)	0.072
Female, n (%)	1253 (58.0)	569 (45.7)	<0.001	1712 (55.0)	196 (45.0)	<0.001	1737 (54.0)	155 (50.2)	0.425
Ethnicity, n (%)			<0.001			<0.001			<0.001
Chinese	1682 (77.8)	636 (51.0)		1957 (62.9)	385 (88.3)		2117 (65.8)	244 (79.0)	
Malay	262 (12.1)	318 (25.5)		645 (20.7)	24 (5.5)		593 (18.4)	38 (12.3)	
Indian	218 (10.1)	292 (23.4)		511 (16.4)	27 (6.2)		507 (15.8)	27 (8.7)	
Education, n (%)			<0.001			<0.001			<0.001
Primary and below	607 (28.1)	259 (20.8)		821 (26.4)	97 (22.2)		887 (27.6)	44 (14.2)	
Secondary	875 (40.5)	581 (46.6)		1352 (43.4)	179 (41.1)		1404 (43.6)	120 (38.8)	
Polytechnic and diploma	339 (15.7)	207 (16.6)		494 (15.9)	73 (16.7)		495 (15.4)	59 (19.1)	
University	341 (15.8)	199 (16.0)		446 (14.3)	87 (20.0)		431 (13.4)	86 (27.8)	
Alcohol consumption, n (%)			<0.001			<0.001			<0.001
Non-drinker	1772 (82.0)	104 (83.5)		2623 (84.3)	319 (73.2)		2683 (83.4)	232 (75.1)	
<1 serving per day	342 (15.8)	185 (14.9)		438 (14.1)	104 (23.9)		474 (14.7)	66 (21.4)	
≥1 serving per day	48 (2.2)	21 (1.7)		52 (1.7)	13 (3.0)		60 (1.9)	11 (3.6)	
Cigarette Smoking, n (%)			0.397			0.093			0.389
Non smoker	1745 (80.7)	976 (78.3)		2493 (80.1)	347 (79.6)		2560 (79.6)	250 (80.9)	
Ex smoker	164 (7.6)	120 (9.6)		246 (7.9)	40 (9.2)		267 (8.3)	30 (9.7)	
Current (<10/day)	77 (3.6)	50 (4.0)		113 (3.6)	18 (4.1)		118 (3.7)	9 (2.9)	
Current (≥ 10/day)	176 (8.1)	100 (8.0)		261 (8.4)	31 (7.1)		272 (8.5)	20 (6.5)	
Coffee (cups/week) [b]	9.0 ± 8.3	7.0 ± 7.7	<0.001	8.3 ± 8.1	7.8 ± 7.9	0.277	8.4 ± 8.1	7.4 ± 8.2	0.003
Chinese Tea (cups/week) [b]	2.0 ± 5.7	2.2 ± 4.9	<0.001	0.1 ± 0.2	14 ± 8.9	<0.001	1.4 ± 4.5	6.6 ± 8.0	<0.001
Green Tea (cups/week) [b]	1.4 ± 4.1	1.8 ± 3.9	<0.001	1.0 ± 3.4	4.6 ± 6.1	<0.001	0.1 ± 0.1	12.6 ± 6.9	<0.001
Black tea	0.1 ± 0.1	12 ± 5.5	<0.001	4.3 ± 6.2	4.8 ± 6.0	<0.001	4.1 ± 6.0	5.3 ± 7.1	<0.001

(cups/week) [b]									
Physical activity (kcal/wk)	3641.0 (1870.9-6564.4)	4001.2 (2082.7-7495.1)	0.001	3744.6 (1947.8-6852.2)	3824.6 (2012.9-7204.0)	0.094	3730.4 (1915.6-6866.3)	3819.4 (1952.2-6605.7)	0.600
% energy from SFA	10.7 ± 2.9	11.6 ± 2.9	<0.001	11.1 ±2.9	10.7 ± 2.9	<0.001	11.1 ±2.9	10.8 ±2.6	0.237
% energy from MUFA	9.7 ±2.7	9.6 ± 2.6	0.035	9.6 ± 2.6	9.7 ± 2.7	0.007	9.6 ±2.6	9.9 ±2.7	<0.001
% energy from PUFA	5.4 (4.2-7.4)	5.2 (4-7.2)	0.003	5.3 (4.1-7.4)	5.3 (4.2-7)	0.188	5.3 (4.1-7.2)	5.9 (4.4-7.7)	0.008
Fiber (g per 1000 kcal)	10.7 (9.2-12.4)	10.4 (9.0-12.2)	0.019	10.5 (9.1-12.3)	10.3 (8.8-12.5)	0.355	10.5 (9.1-12.3)	10.6 (9-12.6)	0.351
Cholesterol (mg per 1000 kcal)	113.5 (88.6-140.5)	112.1 (85.7-139.2)	0.056	112.8 (86.9-139.9)	111.2 (89.7-143)	0.124	112.1 (86.7-139.5)	112.1 (88.6-137.0)	<0.001
History of hypertension, n (%)	380 (17.6)	229 (18.4)	0.026	517 (16.6)	106 (24.3)	<0.001	558 (17.4)	72 (23.3)	<0.001
History of dyslipidemia, n (%)	562 (26.0)	369 (29.6)	0.061	836 (26.9)	133 (30.5)	0.209	870 (27.0)	100 (32.4)	0.135

[a] Abbreviations: SFA; saturated fatty acid, MUFA; monounsaturated fatty acids, PUFA; polyunsaturated fatty acids. Data are presented as mean ± SD, median (IQR) or n (%) unless otherwise specified

[b] Median values were assigned to each category of coffee/tea as follows: never/rarely;0, < 1 cup/week; 0.4, > than 1 cup/wk but < 1cup/d; 4;1-2 cups/d ; 10.5; 3-5 cups/d ; 28; 6-9 cups/day; 52.5 and 10 or more cups/day; 70. Means and SD are presented in the tables; due to skewed data the Kruskal-Wallis test was used to test for differences between categories.

Table S2: Geometric means (95% CI) of glycemic and inflammatory parameters by categories of total tea consumption [a]

	Never or rarely	<1 cup per day	1 to < 3 cup(s) per day	≥3 cups per day	*P* trend [b]
Fasting plasma glucose (mmol/L)					
Model-1 [c]	4.87 (4.83 - 4.91)	4.83 (4.79 - 4.86)	4.83 (4.8 - 4.86)	4.85 (4.79 - 4.91)	0.660
Model-2	4.78 (4.72 - 4.85)	4.75 (4.69 - 4.81)	4.75 (4.69 - 4.81)	4.75 (4.68 - 4.83)	0.360
HOMA-IR					
Model-1	1.55 (1.48 - 1.62)	1.5 (1.44 - 1.57)	1.58 (1.53 - 1.64)	1.67 (1.56 - 1.78)	0.015
Model-2	1.42 (1.33 - 1.52)	1.39 (1.3 - 1.48)	1.42 (1.34 - 1.51)	1.42 (1.31 - 1.53)	0.796
HOMA-beta					
Model-1	101.09 (97.51 - 104.8)	103.22 (99.53 - 107.05)	103.57 (100.52 - 106.7)	103.57 (98.08 - 109.37)	0.445
Model-2	103.94 (97.39 - 110.93)	105.89 (99.31 - 112.91)	105.87 (99.62 - 112.52)	104.96 (97.24 - 113.29)	0.800
HbA1c %					
Model-1	5.8 (5.76 - 5.83)	5.76 (5.73 - 5.8)	5.78 (5.75 - 5.81)	5.81 (5.76 - 5.87)	0.411
Model-2	5.74 (5.68 - 5.8)	5.72 (5.66 - 5.78)	5.72 (5.66 - 5.78)	5.74 (5.67 - 5.81)	0.884
Adiponectin : high-molecular weight (µg/ml)					
Model-1	1.04 (0.99 - 1.1)	1.04 (0.99 - 1.09)	1.02 (0.98 - 1.06)	0.96 (0.89 - 1.03)	0.039
Model-2	1.01 (0.93 - 1.1)	1.01 (0.93 - 1.09)	1.02 (0.94 - 1.1)	0.99 (0.9 - 1.09)	0.650
CRP (mg/L)					
Model-1	1.53 (1.42 - 1.65)	1.42 (1.31 - 1.53)	1.49 (1.4 - 1.58)	1.55 (1.39 - 1.74)	0.590
Model-2	1.41 (1.25 - 1.59)	1.32 (1.17 - 1.48)	1.36 (1.21 - 1.52)	1.31 (1.14 - 1.51)	0.388

[a] To compute total weekly tea consumption, each category was assigned a median value as follows: never or rarely (0), < 1 cup per week (0.5), More than 1 cup per week but less than 1 cup per day (4), 1-2 cups per day (10.5), 3-5 cups per day (28), 6-9 cups per day (52.5) and 10 or more cups per day (70). This was added for the 3 types of tea. HOMA-IR, homeostatic model assessment-insulin resistance; HOMA-beta, homeostatic model assessment-beta cell function; CRP, C-reactive protein. Numbers of participants differ across outcomes because participants with outlier values (response values > 4 SD from mean) were excluded. Numbers were as follows: fasting plasma glucose: 1050, 1085, 1515, 424; HOMA-IR: 1057, 1093, 1531, 427; HOMA-beta: 1057, 1090, 1527, 421; HbA1c: 849, 887, 1246, 371; HMW adiponectin: 1028, 1061, 1508, 420; and CRP: 1022, 1054, 1502, 417.

[b] *P*-values were obtained from multiple linear regression models with median cups of tea per week (0, 4,10.5, 28) as predictors and log transformed parameters as dependant variables.

[c] Model-1: adjusted for age (years), sex and ethnicity (Chinese, Malay, Indian).

Model-2: adjusted for Model 1 covariates (above) and BMI (kg/m^2), physical activity level (kcal/week), education level (primary, secondary, polytechnic/diploma and university), alcohol level (non-drinkers, <1 serving/day, and ≥ 1 serving/day), cigarette smoking (never-smokers, ex-smokers, current smokers <10 cigarettes /d, and current smokers ≥ 10 cigarettes/d), history of dyslipidemia (yes/no), history of hypertension (yes/no), and dietary confounders i.e. energy intake (kcal), fiber (per 1000 kcal), cholesterol (per 1000 kcal), PUFA (% energy), MUFA (% energy), SFA (% energy) and

coffee (never/rarely, < 1 cup per day, 1-2 cup(s) per day, ≥ 3 cups /day) . HOMA-beta models were further adjusted for HOMA-IR .
